# Teleost Chemokines and Their Receptors

**DOI:** 10.3390/biology4040756

**Published:** 2015-11-11

**Authors:** Steve Bird, Carolina Tafalla

**Affiliations:** 1 Biomedical Research Unit, School of Science, University of Waikato, Waikato 3240, New Zealand; E-Mail: sbird@waikato.ac.nz; 2 Animal Health Research Center (CISA-INIA), Carretera de Algete a El Casar km. 8.1, Valdeolmos, Madrid 28130, Spain

**Keywords:** fish, teleost, chemokines, ligands, receptors

## Abstract

Chemokines are a superfamily of cytokines that appeared about 650 million years ago, at the emergence of vertebrates, and are responsible for regulating cell migration under both inflammatory and physiological conditions. The first teleost chemokine gene was reported in rainbow trout in 1998. Since then, numerous chemokine genes have been identified in diverse fish species evidencing the great differences that exist among fish and mammalian chemokines, and within the different fish species, as a consequence of extensive intrachromosomal gene duplications and different infectious experiences. Subsequently, it has only been possible to establish clear homologies with mammalian chemokines in the case of some chemokines with well-conserved homeostatic roles, whereas the functionality of other chemokine genes will have to be independently addressed in each species. Despite this, functional studies have only been undertaken for a few of these chemokine genes. In this review, we describe the current state of knowledge of chemokine biology in teleost fish. We have mainly focused on those species for which more research efforts have been made in this subject, specifically zebrafish (*Danio* *rerio*), rainbow trout (*Oncorhynchus* *mykiss*) and catfish (*Ictalurus* *punctatus*), outlining which genes have been identified thus far, highlighting the most important aspects of their expression regulation and addressing any known aspects of their biological role in immunity. Finally, we summarise what is known about the chemokine receptors in teleosts and provide some analysis using recently available data to help characterise them more clearly.

## 1. Introduction

Chemokines or chemoattractant cytokines are a family of cytokines that regulate immune cell migration under both inflammatory and normal physiological conditions. Constitutively expressed chemokines regulate homing, maturation and even microenvironmental segregation of immune cells within lymphoid organs [1,2]. Outside the immune system, these molecules have also shown to have a role in angiogenesis [3,4], neurological development and function [5,6], organogenesis and germ cell migration [7,8,9]. In response to a pathogenic exposure, chemokines not only promote leukocyte mobilization, but also regulate the immune responses and differentiation of the recruited cells to orchestrate the first steps of both innate and acquired immune responses [10,11].

Chemokines are defined by the presence of four conserved cysteine residues and are divided into four subfamilies based on the distinctive pattern of the two N terminal cysteines: CXC (α), CC (β), C and CX_3_C classes [12]. In mammals, the CXC and the CC families are the two largest, with multiple members in each, whereas the CX_3_C and C families only contain one and two members respectively. In fish, this proportion is still maintained and no CX_3_C chemokines have ever been reported whereas C chemokines have only been reported thus far in zebrafish (*Danio* *rerio*) [13]. In this species, a further fish-specific chemokine subfamily has been identified and designated as CX. These CX chemokines lack one of the two N-terminus conserved cysteine residues but retain the third and the fourth ones in contrast to the C family that only retains the second and fourth of the signature cysteine residues [13].

In mammals, chemokine receptors interact with the members of each subfamily, allowing the co-ordination of the ligands activities. Each receptor belongs to the largest rhodopsin family of G-protein coupled receptors (GPR), structurally consisting of seven transmembrane domains, with multiple extracellular and intracellular loops involved in ligand binding and signalling [14]. Chemokine receptors are classified into subgroups, depending on the ligands they bind and are either, CXC chemokine receptors (CXCR), CCR, XCR or CX3CR [15,16]. In addition, a new group of receptors that scavenge ligands, suppressing chemotactic responses are also found and called, atypical chemokine receptors (ACKR) [17,18].

In mammals, CXC chemokines can be further divided into two main groups depending on whether or not they contain an ELR (Glu-Leu-Arg) motif at the N-terminus of their sequence. In mammals, this motif is responsible for receptor binding and activation of neutrophils, whereas CXC chemokines that lack this motif do not attract neutrophils and act on monocytes and lymphocytes [19,20]. There are eight human ELR^+^ CXC chemokine genes (CXCL1–3, 5–8, 15) known to act through receptors CXCR1 and CXCR2, and nine ELR^−^ CXC chemokines that interact with receptors CXCR3-6 [21,22]. In fish, this ELR motif is usually replaced by a defective DLR motif (Asp-Leu-Arg) thought at first to be active due to the fact that mammalian ELR motifs mutated to DLR retained the capacity to attract neutrophils [23]. However, it has been recently demonstrated that this DLR motif is not essential for the attraction of neutrophils by fish CXC chemokines, and therefore DLR fish chemokines attract neutrophils even when this motif is eliminated [24].

In 1998, Dixon *et* *al*. reported the first chemokine gene in teleost fish [25]. This CC chemokine identified in rainbow trout (*Oncorhynchus* *mykiss*) was designated as CK1. Since then, many fish chemokine sequences have been identified, due to the recent progress in genomic sequencing achieved in selected species. However, teleost fish are a highly diverse group which constitutes approximately one half of the known vertebrate species. Consequently, chemokines which are known to evolve more quickly than other immune genes, being one of the eight most rapidly changing proteins as a reflection of different infectious experiences [26,27], are highly divergent in these different teleost species. This is supported by the discovery that the repertoire of chemokines, especially CC chemokines, is larger in fish than in mammals and includes a large group of fish-specific chemokines. In addition, characterization of the actual chemokine receptors present in teleosts has begun, but again the actual repertoire in fish, compared to mammals is not entirely clear. All this, makes it very difficult to establish true orthologues between fish and mammals and even between the different fish species. Consequently, no clear inferences as to the chemokine functions have been obtained based on their similarities to either potential mammalian counterparts or fish homologues, and the functionality and regulation of each fish chemokine identified and the receptor they may bind, have to be experimentally addressed.

Throughout this review, we have outlined the chemokine genes that have been identified so far in teleost fish, specifically focusing on species such as zebrafish, rainbow trout, catfish (*Ictalurus* *punctatus*) and carp (*Cyprinus* *carpio*) in the case of CXC chemokines, since it is in these species where more research efforts have been made from an immunological point of view. Because it would be desirable to undertake a collective effort to unify fish chemokine nomenclature, as that previously performed in mammals [12], we also discuss previous attempts to classify fish chemokine genes and highlight the most important aspects of their transcriptional regulation and immune functionality for those chemokines for which functional assays have been performed. Finally, we discuss what is currently known about chemokine receptors present in teleosts and use all available data to provide a much clearer summary of what is actually present in this group of vertebrates.

## 2. Identification, Transcriptional Regulation and Functionality of Teleost CC Chemokine Genes

### 2.1. Zebrafish CC Chemokines

An initial study using expressed sequence tags (EST) resources as well as the zebrafish draft genome sequence identified a total of 46 putative CC chemokine genes in zebrafish. The majority of these genes appeared to be derived from local duplication events, which were found to be species-specific when compared to data from another three fish species [28]. That same year, further analysis of the draft genomes of several species to systematically identify chemokines and chemokine receptors reported some additional CC chemokine genes [9]. Finally, two years later, a total of 111 chemokine genes had been detected [13], 81 of which belonged to the CC family. This was a very surprising result at the time as fish had been expected to possess a far simpler chemokine system than mammals. For those chemokine genes that could not be recognized as true orthologues of mammalian genes, zebrafish chemokine genes were designated according to their subfamily (CCL, CXCL, XCL, or CXL) together with an L standing for ligand, and followed by the chromosome number prefixed with chr and by alphabets to distinguish individual genes on a given chromosome [13].

Despite the high number of CC chemokine genes identified in zebrafish, very few expression studies have been performed for these molecules. The transcription of 14 CC chemokine genes was studied throughout the embryonic development by Nomiyama *et* *al*. [13] finding that the transcription of most of the examined chemokine genes started at specific embryonic stages and continued to increase up until six months after fertilization. In contrast, CCL-chr24a was exclusively expressed at early larval periods whereas other chemokines such as CCL-chr25s, CCL-chr20d, and CCL-chr5b were expressed mainly in adults. While these results suggest that the chemokines examined might have a role during zebrafish development, no further studies concerning their regulation during an immune stimulation were included in these studies. On the other hand, a dual-colour RT-MLPA (reverse transcription—multiplex ligation-dependent probe amplification) method used to analyse the transcription of 34 different genes in *Mycobacterium* *marinum*-infected zebrafish adults and *Salmonella* typhimurium-infected embryos [29] included the analysis of CCL-chr24i, CCL-chr5a and CCL20 amongst the genes analysed. The results obtained revealed a marked up-regulation of CCL-chr24i and CCL-chr5a in response to both infection models. Finally, the distribution of a chemokine identified initially as a mammalian CCL21 homologue, was studied both in zebrafish embryos and in adult tissues [30]. However, a recent and more extensive phylogenetic analysis, has suggested that this sequence may not in fact, be a true CCL21 homologue, proposing that teleost fish, may lack CCL21 [31]. Despite its true identity, using *in* *situ* hybridization, transcripts of this gene were observed in the craniofacial region, pharyngeal region, and blood vessels in early embryonic stages of zebrafish whereas in adults, expression was observed in the spinal cord, kidney, and a percentage of blood cells. The fact that this gene is expressed in blood vessels during embryonic development led the authors to speculate a role for this chemokine in angiogenesis [31]. Lastly, CCL25 has also been characterized in fish, where two genes have been reported in both zebrafish [32] and medaka (*Oryzias* *latipes*) [33] and their expression levels in the thymus have been extensively studied throughout development [30]. In mammals, CC chemokines are chemoattractant for mononuclear cells, namely monocytes/macrophages and different lymphocyte subtypes specific to each molecule [34]. In zebrafish, the chemoattractant capacity of the CC chemokines has only been indirectly addressed for CCL25 [32], since morpholino-mediated knockdown of CCL25a in this species interfered with the normal recruitment of T lymphocytes into the thymus, while the knockdown of CCL25b had no significant effect.

### 2.2. Rainbow Trout CC Chemokines

In rainbow trout, after the designation of the first CC chemokine as CK1 [25] this nomenclature was maintained for subsequently identified CC chemokine genes such as CK2 [35], CK3 (EMBL Accession number AJ315149) and 15 new rainbow trout CC chemokine sequences identified using available EST databases [34]. A CC chemokine gene, designated as CCL4 was also reported around the same time, however this gene corresponds to what was termed CK5B following the customary nomenclature [36]. Five of the trout CC chemokines are represented by two sequences and have been designated as variants of the same gene (A and B) [34], but even though they share very high identity, each variant is differently regulated [34,37].

Concerning their transcriptional regulation, CK5B and CK6 mRNA levels were shown to increase in the RTS11 macrophage cell line upon stimulation with tumour necrosis factor α (TNF-α) [34]. Additionally, CK5B was also proven to be highly regulated by LPS since the *in* *vivo* administration of LPS provoked significant up-regulations of CK5B mRNA levels in intestine, ovaries and spleen along with a down-regulation of its transcription in gills [36]. Concerning their regulation in response to viral infections, a group of chemokines (CK1, CK3, CK5B, CK6, CK7A, CK9 and CK12) were selected to study the effects provoked by an intraperitoneal injection with viral haemorrhagic septicaemia virus (VHSV) or infectious pancreatic necrosis virus (IPNV) on their levels of transcription in the spleen and head kidney [38,39]. The results showed important differences in the chemokine profile induced by each pathogen, with VHSV modulating CK1, CK3, CK5B, CK6 and CK12 or IPNV affecting CK1, CK5B, CK6, CK7A, CK9 and CK12. The chemokine profiles not only differed between the two pathogens but a difference in tissue-specific expression was also demonstrated in response to each specific pathogen. Likewise, the response to VHSV when fish were infected by bath immersion was studied in the fin base area (the main portal of rhabdovirus entry) and the gills [40]. In this case, among all the rainbow trout chemokine genes studied, only the transcription levels of CK10 and CK12 were significantly upregulated in response to VHSV at the fin base. A significantly stronger chemokine response was triggered conversely in the gills, with CK1, CK3, CK9, and CK11 being upregulated in response to VHSV and CK10 and CK12 down-regulated by the virus. Because active viral replication was taking place at the fin base but not at the gills, these results suggested a VHSV interference mechanism on the early chemokine response at its active replication as a possible key process that may facilitate viral entry. In the case of IPNV, an additional study revealed that CK9, CK10, CK11 and CK12 are up-regulated in different segments of the digestive tract upon an experimental bath infection [41]. It should be noted that the modifications in the transcription levels of different chemokine genes observed in response to immune stimulation in *in* *vivo* experiments could be a consequence of variations in the levels of mRNA produced by resident cells in the tissue or due to changes in the cell types responsible for their production.

Regarding the role that these molecules have during vaccination, our group also demonstrated that intramuscular vaccination of rainbow trout with a VHSV DNA vaccine significantly induced the transcription of CK5A, CK5B, CK6, CK7A and CK7B in head kidney [37] with CK5B and CK6 increased in the muscle area surrounding the injection site [42]. In the digestive tract, the oral administration of an alginate-encapsulated IPNV DNA vaccine provoked the transcriptional up-regulation of CK9, CK10, CK11 and CK12 although differences were observed along the different gut segments for each of them [41].

In rainbow trout, recombinant CK1 has been shown to be an attractant for blood leukocytes [25]. However, recombinant CK6 is a chemoattractant for mature macrophages from the RTS11 rainbow trout monocyte-macrophage cell line [43]. In addition, it was demonstrated that CK6 was capable of inducing interleukin 8 (IL-8), inducible nitric oxide synthase (iNOS) and the CD-18 integrin in these cells, revealing additional immunomodulatory effects. The capacity of trout recombinant CK12 to attract splenocytes has also been reported, establishing that IgM^+^ B cells were one of the target cells recruited [44].

### 2.3. Catfish CC Chemokines

In 2004, 14 CC chemokines were identified in channel and blue catfish by analysis of ESTs [45]. As occurred in other fish species, because no clear orthologies with mammalian CC chemokines could be established, these genes were designated as SCY (small inducible cytokines) followed by the letter A previously used to designate the CC family of chemokine genes [12]. Because mammalian chemokines are known to be highly clustered in the genome, the previously identified CC chemokines were used to map additional genes within bacterial artificial chromosome (BAC) clones. Through this methodology, 12 novel CC chemokine genes were identified, bringing the total to 26 [28].

Concerning expression analysis, out of the 26 catfish CC chemokine genes, 14 were universally expressed in spleen, liver, head and trunk kidney, skin, stomach, intestine, gills and ovary; six were widely expressed in many tissues, and the other six were highly tissue-specific. Among these tissue-specific chemokines, for example, SCYA118 was only detected in the intestine; SCYA109 was only detected in the gills; and SCYA126 only in gills and skin [46]. Interestingly, SCYA126 is closely related to the mammalian CCL27 known to play a major role in T cell homing to the skin [47] and rainbow trout CK11 that is also majorly expressed in gills and skin [40]. The effects that a bacterial infection with *Edwardsiella* *ictaluri* had on the levels of transcription of these chemokines in spleen and head kidney were also determined [28] finding that seven out of the 26 CC chemokines analysed were upregulated in response to *E*. *ictaluri*, namely SCYA105, SCYA109, SCYA112, SCYA113, SCYA115, SCYA117, and SCYA125. Conversely, SCYA116 and SCYA121 were down-regulated in response to the bacterial challenge. To date, no functional studies have been undertaken with catfish CC chemokines.

### 2.4. CC Chemokines in Other Fish Species

There are a number of other fish species, mainly of interest to the aquaculture industry, for which CC chemokine sequences have been identified and studies dealing with the regulation of their expression performed. For gilthead seabream (*Sparus* *aurata*), one of the main cultivated species in the Mediterranean Sea, one partial CC chemokine sequence designated as CCL4 (Accession no. AM765840) was first deposited in GenBank. Additionally, in 2010, our group identified six sequences within EST seabream databases corresponding to novel CC chemokine genes [48]. We named these genes according to the rainbow trout CC chemokines with which they had the highest identity, CK1, CK3, CK5, CK7, CK8 and CK10. In addition, after analysis of all available sequences, it was proposed that the previously identified seabream chemokine designated as CCL4, should be renamed CK5B. The effect that different immune non-replicative stimuli had on the levels of expression of each of these chemokines was studied in head kidney leucocytes. Although most of these stimuli provoked strong suppressive effects on the transcription levels of these chemokines, up-regulations of mRNA levels were observed in response to mitogens. *In* *vivo*, when non-replicative virus particles were injected, chemokine transcription was induced in the spleen but not in head kidney. Finally, in the context of an experimental infection with nodavirus, all the CC chemokines studied were significantly induced in the brain, suggesting an important role for these chemokines in the recruitment of leukocytes to major replication sites during the course of viral infections.

In Japanese flounder (*Paralichthys* *olivaceus*), at least six different CC chemokine genes have been reported and their involvement in immune regulation during the course of a pathogenic process demonstrated using different infection models [49,50,51,52]. The chemoattractant potential of two of them, Paol-SCYA104 [50] and JFCCL3 [51], has also been demonstrated after the production of the recombinant proteins using purified blood leukocytes. In orange-spotted grouper, a homologue to mammalian CCL4 was reported [53]. Recombinant CCL4 was found to have chemotactic activity for peripheral blood leukocytes [53], as well as the capacity to up-regulate the transcription of TNF-α1, TNF-α2, IFN-γ, Mx, T-bet and both CD8 α and β chains, suggesting that CCL4 not only attracts leukocytes, but also induces an inflammatory response and skews lymphocyte differentiation into the Th1 pathway. In rock bream (*Oplegnathus* *fasciatus*), a CC chemokine, with no specific homology for any mammalian chemokine gene, has also been reported and designated as RbCC1 [54]. Expression was up-regulated in response to mitogen stimulation or pathogen exposure and found to have both chemotactic and proliferative effects on blood leukocytes, indicating an important role in the immune response of this species.

### 2.5. Classification of Teleost CC Chemokines

Mammalian CC chemokines were first divided into “inflammatory” or “inducible” CC chemokines which are expressed only after an immune stimulation and “homeostatic” or “constitutive” CC chemokines which are produced under normal physiological conditions [22]. Similarly, this classification was initially used for fish chemokines [55], however, as more information became available concerning the diverse immune roles of CC chemokines, and many chemokines appeared to have a dual role, this division seemed over-simplistic and was disregarded both in mammals and fish. In 2007, when many of the chemokine genes that we know of today had been identified, Peatman and Liu, established seven large groups of fish CC chemokines through an extensive phylogenetic analysis using CC chemokine sequences from trout, salmon, catfish and zebrafish along with mammalian CC chemokines [27]. These groups were named based on their mammalian membership as the CCL19/21/25 group, the CCL20 group, the CCL27/28 group, the CCL17/22 group, the macrophage inflammatory protein (MIP) group, the monocyte chemotactic protein (MCP) group and a fish-specific group. Because the level of diversity of CC chemokines seems higher in fish than in mammals, and one-to-one orthologous relationships are difficult to establish, this classification into large phylogenetically related groups has been useful to establish some associations between fish CC chemokine genomic locations and evolutionary patterns. It must be noted that a much higher degree of conservation is observed among “homeostatic” CC chemokines reflecting a need to preserve essential physiological functions. To date, no additional attempts to classify fish CC chemokines have been made and consequently this classification is still in use.

## 3. Identification, Transcriptional Regulation and Functionality of Teleost CXC Chemokine Genes

### 3.1. Zebrafish and Carp CXC Genes

In 2008, five CXC chemokines were identified in zebrafish [56]. One of them appeared to be a homologue of CXCL14 whereas three of them had higher homology with CXCL10. Surprisingly, the fifth gene was closest phylogenetically to human CCL25 and catfish CXCL-2-like genes. In that same study, the levels of transcription of these five chemokines was determined in unstimulated fish as well as in fish treated with poly I:C or LPS, revealing a possible immune role for all these chemokines.

Two CXCL8 genes are also present in zebrafish [57,58]. These two genes correspond in fact to two CXCL8 paralogues and have been designated as CXCL8-L1 and CXCL8-L2. Both genes have been shown to be transcriptionally induced in response to wound-associated inflammation [58] and bacterial infection [59]. Interestingly, in some situations, these two CXCL8 are differentially regulated. For example, a recent study has demonstrated that in the intestine CXCL8-L1 but not CXCL8-L2 expression is regulated by T lymphocytes under homeostatic conditions, whereas, during intestinal inflammation, CXCL8-L1 expression is upregulated independent of T lymphocyte presence [60]. This differential expression of the two CXCL8 molecules has also been widely demonstrated in carp, a related cyprinid species in which two CXCL8 paralogues are also present [61]. In mammals, ELR^+^ CXC chemokines, CXCL1-8 (except CXCL4) and CXCL15, are essential for the recruitment of neutrophils to infection sites or tissue injuries [62]. CXCL8 (also named IL-8), a potent neutrophil recruiting chemokine in mammals, is one of the chemokines for which more functional assays have been performed in fish. The chemotactic capacity of both CXCL8 paralogues towards neutrophils has been demonstrated in both zebrafish [57,58] and carp [61]. Additional studies performed in carp [63] further revealed the capacity of CXCL8 to up-regulate the superoxide production of recruited cells. Mammalian CXCL4 and CXCL9-11 chemokines, that lack an ELR motif, act on T cells, dendritic cells and NK through a unique receptor CXCR3 [64]. Surprisingly, this receptor is also expressed in fibroblasts, smooth muscle, epithelial and endothelial cells, where these chemokines produce multiple effects. Overall, seven genes with homology to mammalian CXCL9, -10 and -11 have been identified in the zebrafish genome; however, no transcriptional studies have been performed for these chemokines, commonly designated as CXCb chemokines [65]. Expression studies were undertaken however in carp, where two CXCb genes belonging to the CXCb subfamily were shown to be transcriptionally regulated in response to recombinant interferon γ (IFN-γ) similar to that seen in their mammalian counterparts [65]. Additionally, differences in their sensitivity to LPS and kinetics of CXCb1 and CXCb2 gene expression during zymosan-induced peritonitis were observed, suggesting again a functional diversification of cyprinid CXCb chemokines with functional homology to mammalian CXCL9-11 [65]. Carp CXCb1 showed a strong chemotactic activity towards monocytes, granulocytes and lymphocytes [63]. In zebrafish, two of the IFN-γ-inducible homologues of mammalian CXCL9-11 chemokines have been shown to signal through a homologue of the receptor CXCR3 [66], demonstrating that this chemokine-chemokine receptor pair is conserved in fish. Furthermore, this study implied the existence of a CXCR3-CXCL11 axis in the recruitment of macrophages in response to mycobacterial infection. Interestingly, in this infection model, mutations in CXCR3, limited the macrophage-mediated dissemination of mycobacteria, reducing bacterial load and the associated lesions. Similarly, the fact that CXCR3 and CXCb transcription coincides at the time of inflammation in carp, and that both the CXCb chemokines and the CXCR3 receptor were significantly up-regulated upon IFN-γ stimulation was used to hypothesize that CXCb chemokines are the putative ligands for carp CXCR3 [67].

Two clear orthologues to mammalian CXCL12 have also been reported in zebrafish [68], as well as in carp [69]. Extensive functional studies throughout the developmental stages have demonstrated that zebrafish CXCL12 signals through CXCR4 [68,70,71,72]. In mammals, CXCL12 is chemotactic for lymphocytes and macrophages but has been studied specifically throughout development as it is directly implicated in the migration of hematopoietic cells from fetal liver to bone marrow; in the formation of large blood vessels and in the organization of the nervous system [73]. Studies performed with CXCR4^−^^/−^ mutants in zebrafish have demonstrated that CXCL12 signaling has an essential role in coronary vessel formation by directing migration of endocardium-derived endothelial cells [68]. A role in the development and functionality of the brain in adult fish has also been reported [71], as CXCL12 is essentially expressed in the brain in adults of both zebrafish [71] and carp [69]. Furthermore, in zebrafish it has been demonstrated that even though both CXCL12 genes are also expressed in the thymus, their distribution is slightly different [32]. Lastly, very little is known in fish concerning mammalian CXCL14 which is known to be expressed mainly in mucosal tissues, promoting growth and migration of cells such as lymphocytes, dendritic cells and fibroblasts [74] and for CXCL16 or CXCL17, where only a limited amount of information is currently available.

### 3.2. Rainbow Trout CXC Chemokines

Although several CXCL8 gene variants have been reported in rainbow trout [75], only one CXCL8 lineage with close homology to the CXCL8-L1 paralogue reported in cyprinids seems to be present in this species [76]. Rainbow trout CXCL8 is constitutively expressed in most tissues except the brain and its transcription was stimulated in trout macrophages in response to either LPS or poly I:C [76] and in spleen upon infection with VHSV [77] or infectious hematopoietic necrosis virus (IHNV) [78]. Trout CXCL8 showed chemotactic capacity towards neutrophils [79] and monocyte cells from the RTS11 cell line, in which it provokes the up-regulation of pro-inflammatory cytokines [43].

Concerning chemokines with homology to mammalian CXCL9-11 chemokines, in 2002, one sequence was first reported [80] and designated as γIP or CXCL10. This chemokine was shown to be up-regulated in response to rainbow trout IFN-γ like its mammalian homologue [81] and by poly I:C but not by LPS [80]. This response suggested a role in viral defence as was also established in studies that examined the chemokines response to VHSV infection in which γIP was one of the most expressed chemokines [38,39]. Interestingly, γIP is not induced by IPNV [39], demonstrating a specific regulation. The latest phylogenetic analysis of CXC chemokines performed in fish, has proposed that this chemokine be designated as CXCL11_L1 [82]. Using recombinant IL-1β, type I IFN and IFN-γ, CXCL11_L1 mRNA levels increased in RTG2 and RTS11 cell lines as well as in primary head kidney leukocyte cultures, although differently in each cell type [82]. Interestingly, CXCL11_L1 was one of the three chemokines that was transcriptionally up-regulated at the site of injection in DNA vaccinated fish, along with its potential receptor CXCR3 [42]. Rainbow trout CXCL11_L1 showed chemotactic activity towards head kidney leukocytes. Specifically, the fact that the migrated cells were enriched in CD4 transcripts in comparison to non-migrated populations, suggested that CD4^+^ T helper cells were one of the main attracted cell types [82]. A novel CXCd chemokine family with no homology to mammalian chemokines has also been reported in rainbow trout that includes CXCd1 and a closely related duplicate gene CXCd2. Expression using primers that amplified both family members simultaneously showed up-regulation in the spleen upon challenge with *Yersinia* *ruckeri* [83]. More recently, a third member of this chemokine family has been discovered and a new nomenclature suggested, designating members of this group as CXCL_F1a, CXCL_F1b and CXCL_F1c [82].

A rainbow trout homologue to CXCL14 can be found in public ESTs databases (Accession No. BX311586) and a recent study has demonstrated that, CXCL14 is strongly and specifically up-regulated during oocyte maturation in rainbow trout among a set of genes with pro-inflammatory, vasodilatory, proteolytic and coagulatory functions [84].

As occurs in zebrafish and carp, two CXCL12 molecules are present in rainbow trout (Accession No. HE578135 and HE578136) and thought to have originated from the duplication of a single progenitor gene [82]. Finally, additional CXC chemokines that seem to belong to a fish-specific family have been reported in rainbow trout and designated as CXCL_F2-5 [82]. Transcriptional studies have been performed for CXCL_F4 and CXCL_F5, showing both genes were constitutively expressed in gills, spleen, skin, head kidney, intestine, brain, thymus and liver and could be transcriptionally induced by type I IFN, IL-1β and IFN-γ *in* *vitro* and up-regulated in the head kidney of fish injected with a bacterin [82]. The chemotactic capacity of CXCL_F4 and CXCL_F5 towards head kidney leukocytes has also been established [82].

### 3.3. Catfish CXC Chemokines

Orthologues of mammalian CXCL12 and CXCL14 were reported in catfish in 2005, along with a third CXC chemokine gene for which a clear orthology could be established [85]. These three CXC chemokines genes were constitutively expressed in different catfish, but none of them were regulated in response to an infection with *Edwardsiella* *ictaluri*.

A CXCL8 homologue has also been reported in catfish [86], where up-regulation of its mRNA levels in response to *E*. *ictauri* was reported. Additionally, a CXC chemokine was reported with relatedness to mammalian CXC9-11 chemokines that was also up-regulated in response to an experimental infection with this pathogen [87]. Interestingly, in this study, differential expression profiles were observed between resistant blue catfish and susceptible channel catfish, with blue catfish showing only a modest induction, whereas a drastic elevation of the CXC chemokine levels was observed in channel catfish [87]. To date, no functional studies have been performed with catfish CXC chemokines.

### 3.4. CXC Chemokines in Other Species

CXCL13 plays a central role in guiding B cells to follicles through its cognate receptor CXCR5 [88]. Homologues for CXCL13 have been reported in Japanese flounder [89] and large yellow croaker (*Pseudosciaena* *crocea*) [90], where its chemotactic capacity has been established, showing conservation of CXCL13 in teleosts. In large yellow croaker, the chemokine was constitutively expressed in all tissues except the intestine. Upon induction with polyI:C or an inactivated trivalent bacterial vaccine, CXCL13 gene expression was significantly up-regulated in spleen, head kidney, heart and gills [90]. In Japanese flounder, CXCL13 transcription was induced by recombinant IFN-γ [91].

CXCL8 chemokines have also been reported in multiple fish species. Interestingly, all of these CXCL8 genes lack an ELR motif except those in gadoids such as haddock [92] or Atlantic cod [93]. In Atlantic cod, CXCL8 was strongly induced in the head kidney after the administration of poly I:C or formalin-killed *Vibrio anguillarum* [93]. In response to *V*. *anguillarum*, significant CXCL8 levels were also detected in the intestine, spleen, blood and gills. In haddock, however, CXCL8 was down-regulated in the gills in response to LPS injection whereas no modulation of its mRNA levels was detected in other organs [92]. CXCL8 has also been identified in ayu (*Plecoglossus* *altivelis*), where its capacity to attract monocytes has also been reported [94].

More recently, a orthologue of CXCL12 has been identified in rock bream [95], where upregulation of CXCL12 mRNA expression was demonstrated in the head kidney and spleen during the course of an infection with either bacterial or viral agents, demonstrating that CXCL12 is also involved in immune defense in teleosts.

### 3.5. Classification of CXC Chemokines in Fish

Early phylogenetic analysis of teleost CXC chemokine sequences identified six different teleost CXC chemokine clades: CXCa, CXCb, CXCc, CXCd, CXCL12, and CXCL14 (reviewed in [96]). However chemokines from each clade have not been identified in every species and in the case of rainbow trout for example, only CXCa, CXCb, CXCd members had been reported [83]. With the availability of more CXC ligand sequences from a wider variety of species, due to the availability of genomes, a more unified classification has been proposed, which builds on previous analysis [82]. Clear homologues to mammalian CXCL12, 13 and 14 exist in teleosts. Three fish distinct subgroups, called CXCL8_L1 (previously CXCa), CXCL8_L2 (previously CXCc) and CXCL8_L3 (related to CXC15) exist, that group with mammalian CXCL1-8 and CXCL15. One group, CXCL11_L2 (related to CXCb) exists in teleosts which is related to the mammalian CXCL9, 10 and 11, which are tandemly clustered in the human genome. Four other clades are also apparent, which contain fish specific CXCL genes that had not been included before and have been called CXCL-F2 to F5. Lastly there exist in teleosts additional lineage specific groups of CXC chemokines. CXCL-F1a and CXCL-F1b represents groups that include the known CXCd sequences, however a third relative might also exist, called CXCL-F1c. Using this new nomenclature will allow newly discovered CXCL genes in teleosts to be easily identified and may also lead to new fish lineage specific molecules being discovered as more fish are investigated.

## 4. Identification of Teleost C and Fish-Specific CX Chemokine Genes

Among the 111 chemokine genes identified in the zebrafish genome, only one of them belongs to the C subfamily, also called XC to differentiate it from the CX fish-specific subfamily [13]. However, this chemokine designated as XCL-chr2a, shows little homology to mammalian XC chemokines, and as no transcriptional studies have been reported for this chemokine and no other C chemokine genes have been identified in other fish species, the role of this chemokine subfamily in teleost immunity, remains unknown.

Zebrafish has been shown to possess five additional chemokine genes that retain the third and fourth cysteine residues instead of the second and forth retained in the C subfamily. This novel subfamily only identified to date in this species has been termed as the CX subfamily [13]. Phylogenetic and genome organization analysis showed that zebrafish CX genes have been generated from the CC subfamily after successive tandem duplication events [13]. Among the different CX chemokines, transcriptional studies have only been performed for CXL-chr24a, during embryogenesis, showing a specific expression during a certain period of embryogenesis, leading the authors to suggest an important role in zebrafish development [13]. Furthermore, a strong chemotactic capacity of this chemokine for carp leukocytes was also demonstrated.

## 5. Teleost Chemokine Receptors

With nomenclature for chemokine ligands within non-mammalian vertebrates still being decided on and the discovery of novel molecules in a number of species, it is already clear that the characterisation of chemokines within teleosts present a real challenge to future researchers. However, a number of reviews have begun looking at the chemokine receptors present in teleosts [31,97,98,99,100,101] and unlike the ligands their relationship with known mammalian receptor homologues appears to be well conserved. One approach in unravelling the identity and possible roles of the teleost chemokine ligands would be to identify the actual receptors they bind. In humans there are a number of chemokine receptors that have been discovered and according to the ligands they bind are grouped into CXCR, CCR, XCR CXCR and ACKR subfamilies. A key feature of the chemokine system is the high degree of promiscuity, where a single chemokine can bind several receptors. The chemokine receptors that have been characterised in humans and the ligands they bind have been summarized (Table 1) from two recent reviews [15,16]. Included here are recent discoveries that have identified the receptor for CXCL14 to be CXCR4 [102] and the receptor for CXCL17 to be a new member of the CXCR subfamily, CXCR8 [103]. The following, updates what we know about the chemokine receptor superfamilies and their members within teleosts.

**Table 1:** Summary of the CXCR, CCR, XCR CXCR and ACKR receptor subfamilies found in humans and the ligands they have been shown to bind. In addition to acting as agonists, some chemokines can function as natural chemokine antagonists of other ligand-receptor pairs. Highlighted in green are those receptors where clear orthologues within teleosts has not yet been determined. Highlighted in red are those that appear to have no orthologue in teleosts.

Receptor	Ligand (Agonist)	Ligand (Antagonist)
CXCR1	CXCL6, CXCL7, CXCL8	
CXCR2	CXCL1, CXCL2, CXCL3, CXCL5, CXCL6, CXCL7, CXCL8	
**CXCR3**	CXCL4, CXCL4L1, CXCL9, CXCL10, CXCL11, CXCL13	CCL11
**CXCR4**	CXCL12, CXCL14	
**CXCR5**	CXCL13	
CXCR6	CXCL16	
**CXCR8 (GPR35)**	CXCL17	
CCR1	CCL3, CCL3L1, CCL5, CCL7, CCL8, CCL13, CCL14, CCL15, CCL16, CCL23	CCL26
CCR2	CCL2, CCL7, CCL8, CCL13, CCL16	CCL11, CCL26
CCR3	CCL3L1, CCL5, CCL7, CCL8, CCL11, CCL13, CCL14, CCL15, CCL24, CCL26, CCL28	CXCL9, CXCL10, CXCL11, CCL18
CCR4	CCL17, CCL22	
CCR5	CCL3, CCL3L1, CCL4, CCL5, CCL8, CCL11, CCL13, CCL14, CCL16	CCL7, CCL26, CXCL11
**CCR6**	CCL20, CCL21	
**CCR7**	CCL19, CCL21	
CCR8	CCL1, CCL16, CCL18	
**CCR9**	CCL25	
**CCR10**	CCL27, CCL28	
ACKR1 (DARC)	CCL1, CCL2, CCL5, CCL7, CCL8, CCL11, CCL13, CCL14, CCL16, CCL17, CCL18, CCL22, CXCL1, CXCL2, CXCL3, CXCL4, CXCL5, CXCL6, CXCL7, CXCL8, CXCL9, CXCL10, CXCL11, CXCL13	
**ACKR2 (CCBP2)**	CCL2, CCL3, CCL3L1, CCL4, CCL4L1, CCL5, CCL6, CCL7, CCL8, CCL11, CCL12, CCL13, CCL14, CCL17, CCL22, CCL23, CCL24, CCL26	
**ACKR3 (CXCR7)**	CXCL11, CXCL12	
**ACKR4 (CCR11)**	CCL19, CCL21, CCL25, CXCL13	
ACKR5 (CCRL2)	CCL19	
**ACKR6 (PITPNM3)**	CCL18	
**XCR1**	XCL1, XCL2	
CX3CR1	CCL26, CX3CL1	

### 5.1. CCR Subfamily

In mammals, the CCR chemokine receptor subfamily consists of 10 members, CCR1-10 that specifically bind and respond to cytokines of the CC chemokine family. They are part of a large family of G protein-linked receptors that are known as seven transmembrane proteins, as they span the cell membrane seven times [15,16]. Not many actual CCR sequences have actually been isolated from teleosts and little has been done to characterise them functionally within fish. The first actual receptor sequence isolated was CCR9 from trout [104], which had been initially identified as CCR7, however this had been due to the limited amount of non-mammalian sequences available for comparison at the time of its discovery. Subsequently, there have been a number of other receptors isolated from trout [105], that include a clear orthologue of CCR6, a sequence with similarity to the originally identified CCR9, called CCR9B and a sequence with similarity to CCR3, that appeared to have no specific equivalent in mammals, which has been called CCR13. Expression analysis of each of these genes showed that CCR6 was constitutively transcribed in thymus, gills, hindgut and peripheral blood leukocytes (PBLs), CCR9B was strongly transcribed in thymus and PBLs but also in spleen, gills, hindgut and brain at lower levels. Lastly, CCR13 was strongly detected in spleen, head kidney and PBLs and faintly in thymus, gills, brain and gonad. In the *miiuy* croaker (*Miichthys* *miiuy*), homologues for CCR3 and CCR9 have been isolated [106] and expression analysis showed they were ubiquitously expressed in all tested tissues with their expression significantly upregulated after infection with *V*. *anguillarum* except that of CCR9 in the spleen. CCR9 has also been investigated in seabass that were given recombinant TNF-α as an oral vaccine adjuvant alongside a commercial sea bass oral vaccine against *V*. *anguillarum* [107]. It was found that fish treated with recombinant TNF-α showed a dramatic change of their T lymphocytes distribution and localization in gut mucosal tissue and shown that extravasation and homing of CCR9^+^ T cells in orally vaccinated fish was mediated by CCL25 derived from epithelial cells of the hindgut. This suggests that the CCL25/CCR9 ligand/receptor system may be a conserved feature throughout the vertebrate lineage.

**Figure 1:**
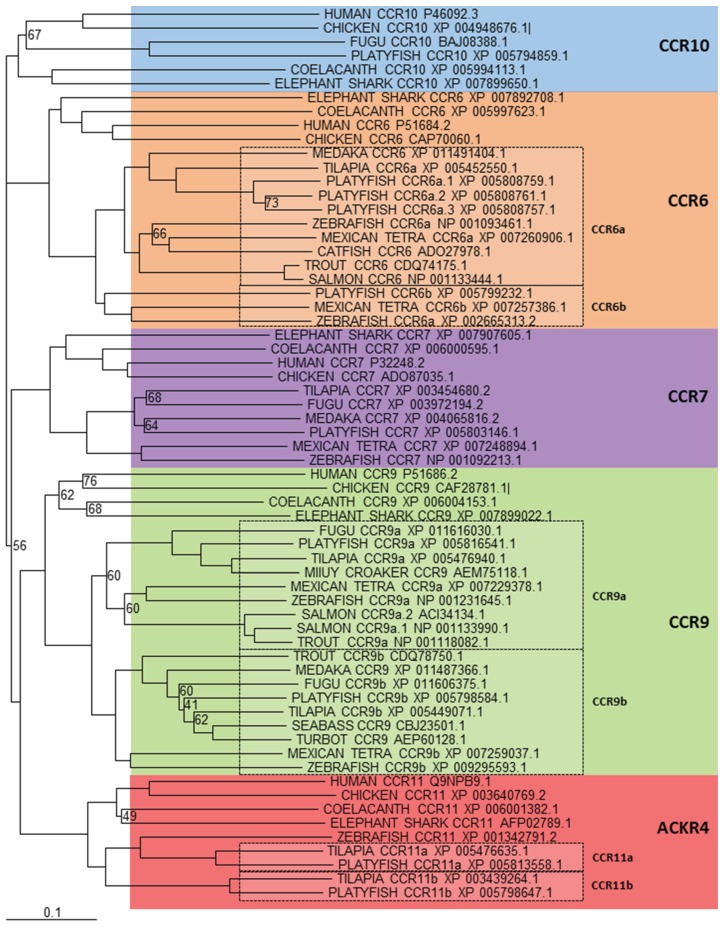
Phylogenetic analysis of human CCR6, 7, 8, 9, 10 and ACKR4 (CCR11) amino acid sequences with identified teleost, reptile, bird, amphibian and cartilaginous fish sequences. Accession numbers of each sequence are included in the figure. Sequences were found by using the FASTA [108] and BLAST [109] suite of programs to search the non-redundant protein sequence database at NCBI. Phylogenetic relationships were constructed from ClustalX v1.81 [110] generated alignments of amino acid sequences using the neighbor-joining method [111], with values <75% shown. The tree was drawn using TreeView v1.6.1 [112] and bootstrapped 1000 times [113]. A different colour is used to indicate the clear clustering of sequences into each receptor group. Boxed regions indicate where there has been an expansion of a particular receptor, within teleosts.

**Figure 2:**
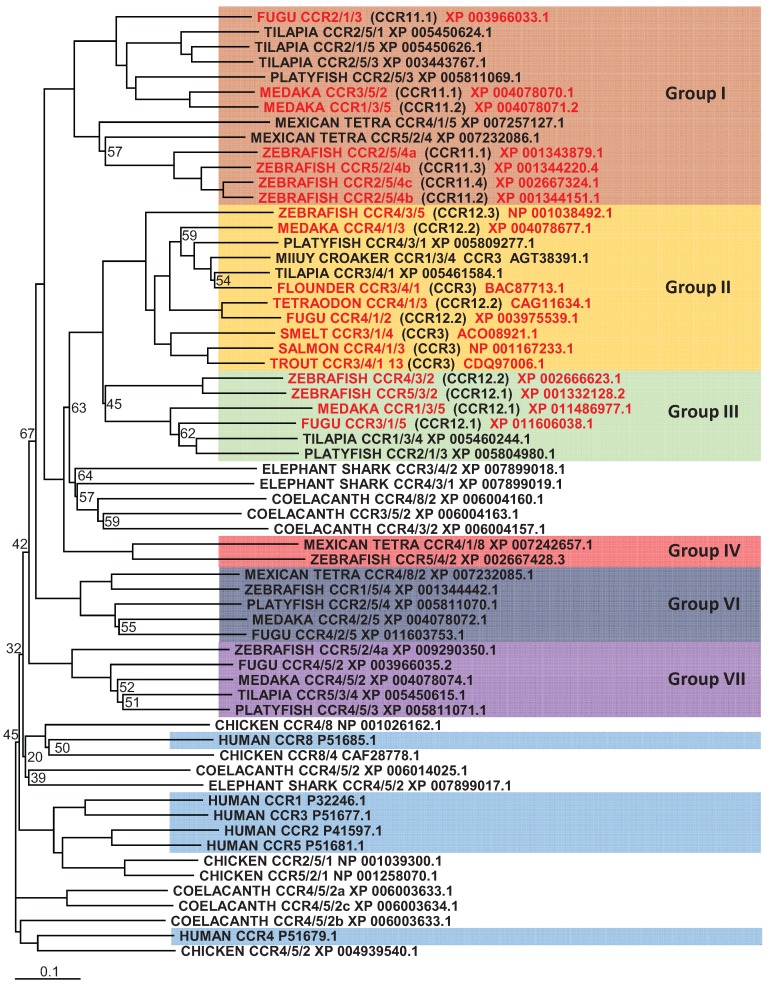
Phylogenetic analysis of human CCR1, 2, 3, 4, 5 and 8 amino acid sequences (highlighted in blue) with identified teleost, reptile, bird, amphibian and cartilaginous fish sequences. Accession numbers of each sequence are included in the figure. Sequences were found and trees generated as described in Figure 1. A different colour is used to indicate the clear clustering of teleost sequences into specific groups. Each non-mammalian sequence was analysed by BLAST and the name reflects the top three mammalian CCR receptors it had highest similarity to. Sequences highlighted in red, indicate those sequences that had been given the name CCR3, CCR11 or 12 in the literature.

In an attempt to characterise the CCR family in teleosts, the zebrafish genome was investigated [97] helping to identify potential orthologs to mammalian CCR6, 7, 8, 9 and 11, however clear orthologs to mammalian CCR1, 2, 3, 4, 5 and 10 were not found. A later investigation looked at genomes from medaka, tetraodon (*Tetraodon* *nigroviridis*) and zebrafish to further characterise the CCR family in teleosts [99]. Here, similar findings were presented, with the addition of two novel teleost receptor groups, named CCR11 and CCR12. This nomenclature is slightly misleading as a new member has been subsequently added to the human subfamily and named CCR11 [114] and has later been included as the atypical chemokine receptor 4 (ACKR4), which has no relationship to the above teleost CCR11. All the information currently available within the databases, allowed us to bring all these discoveries together and analyse them in more detail. Phylogenetic analysis (Figure 1) shows very clearly that orthologues of mammalian CCR6, 7, 9, 10 and ACKR4 (formerly known as CCR11 or CCRL1) exist within teleosts, however in some species more than one gene is found for each representative, indicating certain CC chemokine receptors have expanded in teleost fish. However, not such a clear picture exists for the remaining CCR subfamily members. Included in the phylogenetic analysis (Figure 2) are sequences from a number of different teleost species that bear some resemblance to them, but it is difficult to say whether they are true orthologues or not. The reason for this is that in mammals, these remaining human CC-chemokine receptor genes are very similar in gene structure with CCR1, CCR3, CCR5 and CCR2, clustered on the 3p21.3 region of the human genome and CCR8 and CCR4, spread between this main cluster and the 3p telomere [115,116]. This clearly suggests that all six receptors share a recent common ancestor and originated through gene duplications. Because of the way these genes have evolved in mammals, it appears that a very similar, but separate event has occurred in teleosts, where many genes can be found that bear some resemblance to human CCR1, 2, 3, 4, 5 and 8, however no clear orthologue can be found. Because of this, the teleost CCR3 does not represent a true orthologue and it is too premature to say that teleosts contain novel CCR11, 12 and 13 and it will take a bit more effort to determine the exact nomenclature for these genes in teleosts which will require more investigations to be carried out. What can be seen from the analysis performed is that there are clear groups that these sequences fall into, where fish such as the zebrafish, have clear representatives in each group.

### 5.2. CXCR Subfamily

**Figure 3:**
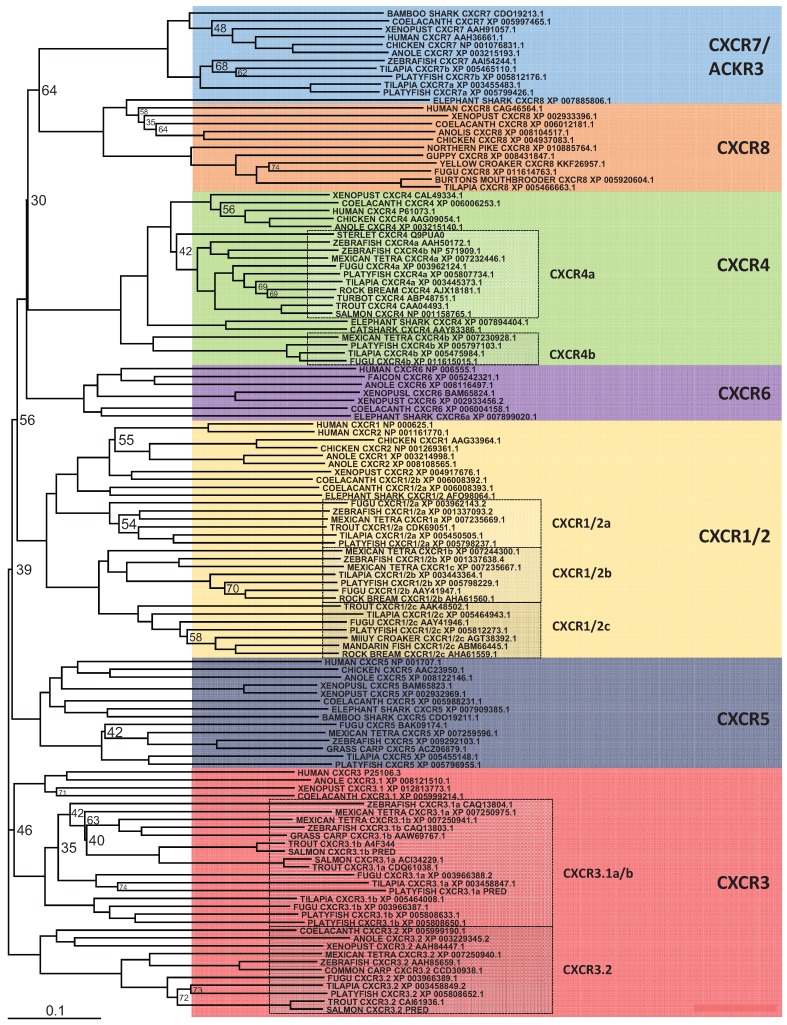
Phylogenetic analysis of human CXCR1, -2, -3, -4, -5, -6, -8 and ACKR3 (CXCR7) amino acid sequences with identified teleost, reptile, bird, amphibian and cartilaginous fish sequences. Accession numbers of each sequence are included in the figure, except where they have been predicted (PRED) using the available genome. Sequences were found and trees generated as described in Figure 1. A different colour is used to indicate the clear clustering of sequences into each receptor group. Boxed regions indicate where there has been an expansion of a particular receptor, within teleosts.

**Figure 4:**
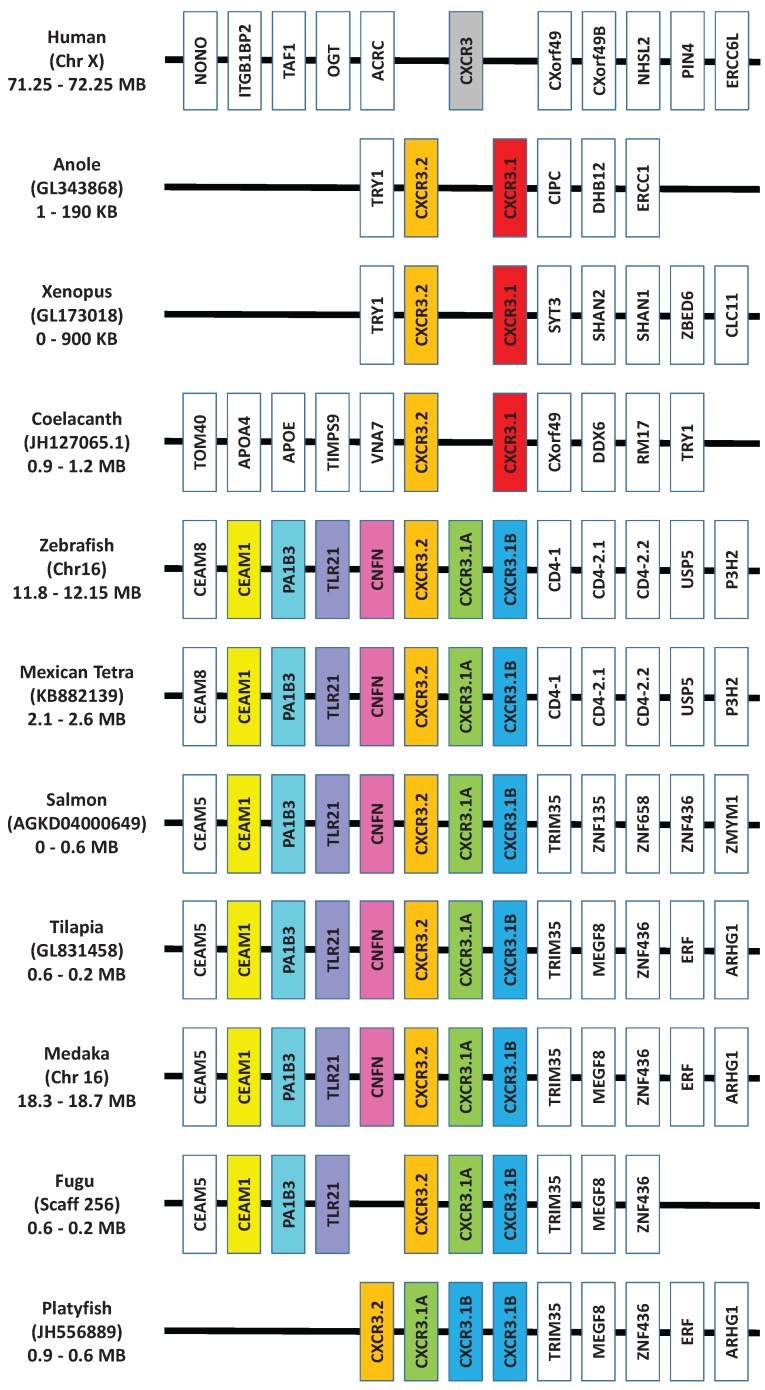
Synteny analysis of the locus containing the CXCR3 gene from human, reptile, amphibian, coelacanth and a selection of teleosts. Genscan [122], BLAST [109] and FASTA [108] were used to analyse the genomes of non-mammalian species to discover the gene order. Three copies of the CXCR3 genes clearly exist in all of the teleost genomes, except platyfish, where four seem to exist.

In mammals, the CXCR chemokine receptor subfamily consists of 8 members, CXCR1-8 that specifically bind and respond to cytokines of the CXC chemokine family and are also part of the large family of G protein-linked receptors known as seven transmembrane proteins [15,16]. In teleosts, a number of CXCR have been isolated from a range of fish species, which has recently been extensively reviewed by Zou *et* *al*. [101]. Human CXCR1-6 and ACKR3 (formerly known as CXCR7) orthologues are shown to be present in cartilaginous fish along with the coelacanth, whereas teleosts are missing CXCR6. In addition, an expansion of a number of the CXC chemokine receptors has occurred in teleost fish. Phylogenetic analysis (Figure 3) also supports the previous findings, however, included in this analysis is a new member of the CXCR subfamily, CXCR8 [103], which is shown to have orthologues in teleosts, as well as cartilaginous fish and the coelacanth. Highlighted in this analysis are the CXCR’s where expansion has occurred in teleosts and there are a number of observations to be made. The naming of CXCR1 and 2 in teleosts, should be more carefully looked at, as it is clear in teleosts that there are three distinct groups of receptors, that are CXCR1/2 like and not a specific orthologue to human CXCR1 or 2. For CXCR3, it had been previously been shown that orthologues could be found in teleosts, and that two apparent groups existed in bony fish, amphibians and reptiles where the genes encoding these reside next to each other in the respective genomes [101,117]. However from looking in more detail at the regions of the available teleost genomes (Figure 4), it can be found that teleosts have an additional CXCR3, not found in amphibians or reptiles that groups more closely with one of the CXCR3 orthologues. Lastly, two very distinct groups can be seen to exist for teleost CXCR4 orthologues. From this it is very clear that there exists in teleosts an expansion of the CXCR receptors that exist in humans, the outcome of which requires much more investigation.

### 5.3. Other Subfamily Members

**Figure 5:**
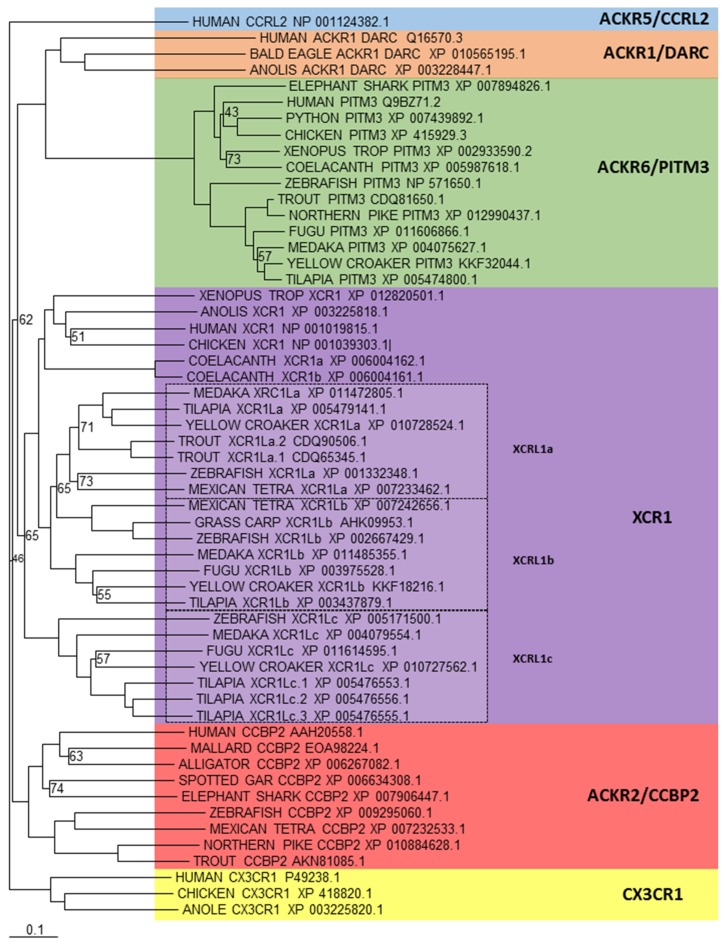
Phylogenetic analysis of human XCR1, CX3CR1, ACKR1 (DARC), ACKR2 (CCBP2), ACKR5 (CCRL2) and ACKR6 (PITPNM3) amino acid sequences with identified teleost, reptile, bird, amphibian and cartilaginous fish sequences. Sequences were found and trees generated as described in Figure 1. A different colour is used to indicate the clear clustering of sequences into each receptor group. Boxed regions indicate where there has been an expansion of a particular receptor, within teleosts.

There are a number of other chemokine receptors that are separate to the CCR or CXCR subfamilies, which includes XCR1 and CX3CR1, which bind XC and CX3C ligands respectively. In addition to all these classical signalling chemokine receptors, there is a new chemokine receptor subfamily in humans that has recently been named ACKR. They structurally resemble conventional chemokine receptors, however they cannot induce directional cell migration, which is the classical response to a chemokine receptor binding to its ligand [17,18]. This is due to their inability to couple G-proteins, which can be partly explained by an altered DRYLAIV motif, leading to an apparent inability to signal, or use alternative signalling pathways to those seen with classical chemokine receptors. Currently there are four members [118,119], ACKR3 (formerly known as CXCR7) and ACKR4 (formerly known as CCR11/CCRL1), which have already been looked at in the above sections, and ACKR1 (formerly known as DARC) and ACKR2 (formerly known as CCBP2). In addition to these, two further receptors have also been identified [114,120] that are being designated ACKR5 (formerly known as CCRL2) and ACKR6 (formerly known as PITPNM3) and are pending confirmation by the International Union of Basic and Clinical Pharmacology Committee on Receptor Nomenclature and Drug Classification (NC-IUPHAR). A recent study, has shown the existence of ACKR2 in teleosts [121] and previous investigations an orthologue of XCR1 [99,100], which appears to have expanded to create a closely related member in teleosts, which has been called XCR1L. Phylogenetic analysis (Figure 5) supports these findings, but also shows that an orthologue to ACKR6 exists in teleosts. In addition, the expansion of XCR1 has created more than just one XCR1L gene in teleosts, having more than three copies in fish, such as tilapia. Lastly, similar to previous investigations, no orthologues can be found for ACKR1, ACKR5 or CX3CR1 within teleosts.

## 6. Conclusions

It is firmly established that in vertebrate immunity chemokines serve a central role through the coordination of immune cell localization and function. In recent years, chemokine research has significantly increased in teleost fish species and whilst early chemokine studies were only investigated in fish model species such as zebrafish in the context of developmental studies, studies are now being performed in aquacultured fish species, focused on immune function and their role in pathogenesis. A great amount of research still has to be performed to completely understand this complex network of molecules, with the exhaustive phylogenetic analyses performed to date showing that a large group of evolutionarily related fish specific chemokines exist and that the chemokine network found in fish is highly specific to each species. Thus, no clear inferences as to the chemokine functions can be made based on their similarities to potential mammalian counterparts and their roles will have to be experimentally addressed. Consequently, more functional efforts to decipher the specific role and regulation of each of these molecules will have to be undertaken in the following years, and possibly identifying receptors for these molecules will be highly beneficial in this task.

It is clear that a large number of mammalian orthologues for the chemokine receptors appear to exist within teleosts. However, it should be noted that many of these receptors have been predicted from available genomes. Whilst this allows us valuable insights into the types of chemokine receptor genes that may be present in fish, it will be important to actually determine the sequence of all genes expressed in all species, as many of the sequences being used are predictions that are not entirely correct or could represent pseudogenes that play no role in the fish immune response. Currently in teleosts, clear orthologues exist for CXCR3, CXCR4, CXCR5, CXCR6, CXCR8, CCR6, CCR7, CCR9, CCR10, ACKR2, ACKR3, ACKR4 and ACKR6, where expansion of a number of these has occurred in teleosts. At present, no orthologues exist for ACKR1, ACKR5, CXCR6 and CX3CR1 and the application of mammalian nomenclature to teleosts is causing confusion in the naming of receptor genes, and true orthologues for teleost CXCR1, CXCR2, CCR1, 2, 3, 4, 5 and 8, which still require further investigation. With the information we now have, it is important to begin looking at the interaction that the known ligands have with their receptors, in order to continue unravelling the chemokine function in teleosts.
